# Development and characterization of influenza M2 ectodomain and/or hemagglutinin stalk-based dendritic cell-targeting vaccines

**DOI:** 10.3389/fmicb.2022.937192

**Published:** 2022-08-08

**Authors:** Titus Abiola Olukitibi, Zhujun Ao, Hiva Azizi, Mona Mahmoudi, Kevin Coombs, Darwyn Kobasa, Gary Kobinger, Xiaojian Yao

**Affiliations:** ^1^Laboratory of Molecular Human Retrovirology, University of Manitoba, Winnipeg, MB, Canada; ^2^Department of Medical Microbiology, Max Rady College of Medicine, Rady Faculty of Health Sciences, University of Manitoba, Winnipeg, MB, Canada; ^3^Centre de Recherche en Infectiologie de l’Université Laval, Centre Hospitalier de l’Université Laval, Québec, QC, Canada; ^4^Special Pathogens Program, National Microbiology Laboratory, Public Health Agency of Canada, Winnipeg, MB, Canada; ^5^Galveston National Laboratory, 301 University Blvd., Galveston, TX, United States

**Keywords:** hemagglutinin stalk, extracellular matrix protein, ebola glycoprotein GP, universal influenza A vaccine, DC targeting

## Abstract

A universal influenza vaccine is required for broad protection against influenza infection. Here, we revealed the efficacy of novel influenza vaccine candidates based on Ebola glycoprotein dendritic cell (DC)-targeting domain (EΔM) fusion protein technology. The four copies of ectodomain matrix protein of influenza (tM2e) or M2e hemagglutinin stalk (HA stalk) peptides (HM2e) were fused with EΔM to generate EΔM-tM2e or EΔM-HM2e, respectively. We demonstrated that EΔM-HM2e- or EΔM-tM2e-pseudotyped viral particles can efficiently target DC/macrophages *in vitro* and induced significantly high titers of anti-HA and/or anti-M2e antibodies in mice. Significantly, the recombinant vesicular stomatitis virus (rVSV)-EΔM-tM2e and rVSV-EΔM-HM2e vaccines mediated rapid and potent induction of M2 or/and HA antibodies in mice sera and mucosa. Importantly, vaccination of rVSV-EΔM-tM2e or rVSV-EΔM-HM2e protected mice from influenza H1N1 and H3N2 challenges. Taken together, our study suggests that rVSV-EΔM-tM2e and rVSV-EΔM-HM2e are promising candidates that may lead to the development of a universal vaccine against different influenza strains.

## Introduction

Influenza is a highly contagious airborne disease that attacks the respiratory system and occurs in pandemics and seasonal epidemics. The influenza pandemic in 1918 killed approximately 50 million people globally (Kedzierska et al., 2018; Nickol and Kindrachuk, 2019), and to date, influenza virus infection is still posing a substantial threat to the health sector worldwide (Valkenburg et al., 2016). The licensed influenza vaccines are associated with some issues, including the level of effectiveness and protection provided by annual vaccines against the specific influenza strain in the seasonal epidemic. Furthermore, there are associated psychological effects on the population who must receive a flu shot every year for their lifetime (Bock et al., 2017; Brewer et al., 2017; Smith et al., 2017). Based on these findings, the Centers for Disease Control, as part of their recent recommendations, emphasizes a need for a universal vaccine against influenza viral infection (Francis et al., 2019).

The universal vaccine is characterized by the ability to protect individuals from different strains of the influenza virus. In addition to the four different types of influenza, each type is composed of a population of different strains (Tong et al., 2013; Francis et al., 2019). Variation in influenza strains occurs in the surface glycoproteins hemagglutinin (HA) and neuraminidase (NA). For instance, influenza A, which is the most prominent family, has 18 known HA subtypes and 11 identified NA subtypes with different host ranges, including humans, birds, bats, and swine (Tong et al., 2013; Francis et al., 2019). The difficulty in producing a universal vaccine against the influenza virus is due to the antigenic shift or antigenic drift caused by reassortment or mutation in HA or NA. These mechanisms allow the influenza virus to continuously escape the host immune defenses (Scorza et al., 2016). Additionally, the currently licensed influenza vaccines are short-lived and show a narrow spectrum (Tsilibary et al., 2021). Therefore, as a method to develop a universal vaccine, the conserved components on the surface proteins of influenza could be utilized to elicit broad immune responses specific to all the present and future strains of the influenza virus.

The influenza HA protein, which is responsible for the cell attachment and entry of the viruses, is a promising epitope in the development of influenza vaccines. Although the globular head of HA induces neutralizing antibodies (Krammer and Palese, 2013; Mullarkey et al., 2016), it contains a large number of mutations and thus cannot be used to develop a universal vaccine. However, the HA stalk and the highly conserved extracellular matrix protein (M2e) are promising for the development of a universal vaccine for influenza viral infection due to their durability and stability (Neirynck et al., 1999; Cummings et al., 2014; Uranowska et al., 2014; Tsybalova et al., 2018). These subunit proteins of influenza induce broader neutralization and participate in either antibody-dependent cellular phagocytosis or antibody-dependent cellular cytotoxicity, which subsequently eliminate the influenza virus or destroy the cells already infected (Mezhenskaya et al., 2019; Von Holle and Moody, 2019). Numerous studies have used different approaches to develop universal influenza vaccines, including fusion of influenza M2e polypeptides (Tsybalova et al., 2018), targeting conserved broadly reactive epitopes on the HA stalk (Krammer et al., 2014), developing fusion proteins between influenza M2e and bacterial flagellin (Turley et al., 2011), expressing recombinant HA in virus-like particles (VLPs; Li et al., 2015), and using VSV to deliver HA antigens (Furuyama et al., 2020). Although some of these approaches are being investigated in clinical trials (Nachbagauer et al., 2020), varying limitations still exist, with the majority having relatively low immune responses, except for VSV or when used with adjuvants, such as MF59 and ASO3 (Scorza et al., 2016). Therefore, new, and efficient universal vaccine(s) with broad protection against various strains of the influenza virus must be developed.

The dendritic cell (DC)-targeting vaccine has recently received global attention, since this approach is effective because DCs function as antigen-presenting cells that stimulate adaptive immune responses and regulate innate immune responses (Brandao et al., 2003). The usage of this technology is in the pipeline for the development of various vaccines against viral pathogens and cancers (Brandao et al., 2003; Banchereau and Palucka, 2005; Querec et al., 2006). A study showed that targeting influenza HA and chemokine receptor Xcr1^+^ to DCs induces immune responses and confers protection against the influenza virus (Gudjonsson et al., 2017). A group of scientists also targeted influenza M2e to DCs by fusing M2e with anti-Clec9 (Park et al., 2017), while HA of influenza was infused with an artificial adjuvant vector cell targeting DCs to induce CD4^+^ T cells and CD4^+^ Tfh cells (Yamasaki et al., 2016).

Ebola virus glycoprotein (EboGP) is the viral protein expressed on the Ebola virus (EBOV) surface that preferentially binds to monocytes, DCs, and macrophages (Martinez et al., 2012, 2013; Olukitibi et al., 2019). As shown in our recent study, the incorporation of EboGP into HIV pseudotyped viral particle (PVPs), indeed, facilitates DC and macrophage targeting and significantly enhances HIV-specific immune responses (Ao et al., 2019). These observations indicated that EboGP has the potential to direct an HIV antigen toward DCs to facilitate effective anti-HIV immune responses. Notably, a highly glycosylated mucin-like domain (MLD) encompassing residues 313 to 501 is located at the apex and the sides of each EboGP monomer (Tran et al., 2014). However, some studies have shown that removing this MLD region does not impede EboGP-mediated lentiviral vector entry (Medina et al., 2003) and was dispensable for EBOV infections *in vitro* (Lee et al., 2008). Our laboratory has recently developed the EboGP MLD replacement system and has shown the great potential of this vaccine technology to deliver heterologous polypeptides *in vivo* and stimulate innate and adaptive immune responses (Ao et al., 2021).

In this study, EboGP DC-targeting domain-based fusion protein technology was used to fuse conserved HA stalk regions (HA stalk) and M2e (HM2e) or four copies of M2e [referred to as tetra M2e (tM2e)] within the MLD-deleted EboGP (EΔM). By incorporating these fusion proteins into HIV-based PVPs or a recombinant vesicular stomatitis virus (rVSV) vector, we characterized their DC/macrophage-targeting ability and investigated their potential for eliciting host immune responses and their abilities to protect against H1N1 and H3N2 influenza infections.

## Materials and methods

### Plasmid construction

**FIGURE 1:**
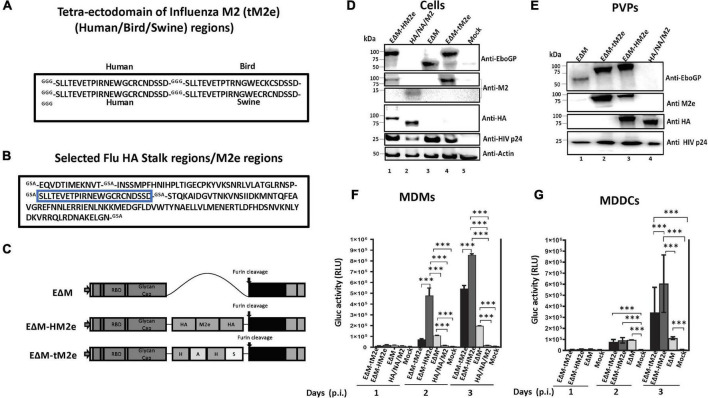
The construction, expression, and cell entry ability of EΔM-tM2e or EΔM-HM2e. **(A)** Amino acid sequences of tM2e, including influenza virus M2e consensus from human, swine, and bird strains. **(B)** Amino acid sequence of HM2e, including influenza virus HA stalk and M2e from human strain. **(C)** EΔM plasmid containing EboGP gene with MLD deletion (aa 305–485) and EΔM-HM2e or EΔM-tM2e plasmids. **(D,E)** 293T cells were co-transfected Δ8.2, ΔRI/ΔE/Gluc+ with EΔM-tM2e and EΔM-HM2e or HA, NA, and M2 plasmids. WB was used to detect the expression of M2e, HA, EboGP, and HIV P24 in cells and PVPs. **(F,G)** Human PBMC-derived macrophages (MDMs) and dendritic cells (MDDCs) were infected with the EΔM-HM2e, EΔM-tM2e, EΔM, and HA/NA/M2-PVPs. The supernatants were collected at different time points after infection and subjected to a GLuc activity assay. Error bars represent variation between duplicate samples, and the data are representative of results obtained in three independent experiments. Statistical significance was determined using an unpaired *t*-test, and significant *p*-values were represented with asterisks ^***^ ≤0.001. No significance (ns) was not shown.

The EboGP with the MLD deletion plasmid (pCAGGS-EΔM) was described previously (Ao et al., 2021). To construct pCAGGS-EΔM-tM2e and pCAGGS-EΔM-HM2e plasmids, we designed and synthesized genes encoding for tM2e [conserved M2e among human (two copies), avian (one copy), and swine (one copy)] as previously described (Liu et al., 2005; aa 92) or HA stalk from H1, H3, H5, and H7 and human M2e (aa 179; HM2e; Figure 1A) and inserted these genes into pCAGGS-EΔM through *Apa*I and *Xba*I sites (Figure 1B). To construct an rVSV vector expressing EΔM-tM2e or EΔM-HM2e plasmids, we inserted the PCR-amplified cDNA encoding a full-EΔM-tM2e or EΔM-HM2e at the *Mlu*I and *Sph*I sites in a VSV-G deleted rVSV vector (Figure 3A). The pCAGGS-HA/NA/M2 plasmids were obtained from H5N1 as previously described (Ao et al., 2008) and the HIV-1 RT/IN/Env tridefective proviral plasmid containing Gaussia gene (ΔRI/ΔE/Gluc) as described previously (Ao et al., 2008).

### Cells, viruses, antibodies, and chemicals

Human embryonic kidney 293T, Vero E6, and Madin-Darby Canine Kidney (MDCK) cell lines were cultured in DMEM supplemented with 10% fetal bovine serum (FBS). To obtain monocyte-derived macrophages (MDMs) or monocyte-derived DCs (MDDCs), human peripheral blood mononuclear cells were isolated from healthy donors by sedimentation on a Ficoll (Lymphoprep; Axis-Shield) gradient and treated with macrophage colony stimulator or granulocyte-macrophage-stimulating factor and interleukin (IL)-4 (R&D system), respectively, for 7 days. Mouse-adapted A/Puerto Rico/8/1934 (H1N1) and 1968 H3N2 (mouse-adapted A/Hong Kong/1/68) used for the animal challenge studies were generated by reverse genetics as previously described (Ranadheera et al., 2018). All viruses were grown and titrated in MDCK cells using the TCID_50_ method.

The M2e monoclonal antibody and HA polyclonal antibody were obtained from Santa Cruz Biotechnology (14C2: sc-32238) and Alpha Diagnostic (HA2H012-A), respectively. Ebola GP monoclonal antibody [(MAb 42/3.7) was kindly given by Dr. A Takada, Hokkaido University, Japan (Takada et al., 2007)]. Human M2e peptide (SLLTEVETPIRNEWGCRCNDSSD) was purchased from GenScript (RP20206), while avian influenza H7N9 M2e peptide (SLLTEVETPTRTGWECNCSGSSD) and swine influenza H1N1 M2e peptide (SLLTEVETPTRSEWECRCSDSSD) were synthesized by GenScript as previously described (Wang et al., 2021) and HA peptide was synthesized by Shanghai Royobiotech (19CL00157). The recombinant HA from H1N1 (11684-V08H), H3N2 (40494-V08B), and H5N1 (40160-V08B1) were obtained from Sino Biological.

### Production and characterization of pseudotyped viral particle or recombinant vesicular stomatitis virus containing EΔM-tM2e and EΔM-HM2e

To produce EΔM-tM2e- or EΔM-HM2e-PVPs for *in vitro* study, 293T cells were co-transfected pCAGGS-EΔM-tM2e or pCAGGS-EΔM-HM2e with Δ8.2 and ΔRI/ΔE/Gluc+. Meanwhile, HA/NA/M2 PVPs were produced by co-transfecting pCAGGS-HA, pCAGGS-NA, and pCAGGS-M2 plasmids with Δ8.2 and ΔRI/ΔE/Gluc+ and used as control. For *in vivo* immunization experiment, 293T cells were transfected with the above plasmids except for ΔRI/ΔE/Gluc+. After 48 h of post-transfection, the VLPs were pelleted by ultracentrifugation at 35,000 rpm (set at the g force of 100,000) at 4°C for 2 h. The virus stocks were quantified by using HIV p24 enzyme-linked immunosorbent assay (ELISA) assay and kept at −80°C.

By using reverse genetics technology (Whitt et al., 2016), the rVSV-EΔM-tM2e or rVSV-EΔM-HM2e vector was transfected into a mix of Vero E6/293T cells together with VSV accessory plasmids encoding for P, L, N, and T7 promoter plasmid. Following the primary transfection, the supernatant containing the recovered rVSV-EΔM-tM2e or rVSV-EΔM-HM2e was amplified in Vero E6 cells. Produced VSV was concentrated by ultracentrifugation, titrated in Vero E6, and used for mice immunization experiments.

To detect the expression and incorporation of M2e, HA stalk and other viral proteins in the transfected cells and PVPs or rVSV were lysed and analyzed by SDS-PAGE and Western blotting (WB) with anti-M2e (14C2), anti-HA, EBOV GP MAb 42/3.7, or anti-HIVp24 antibodies, respectively.

### Growth kinetics

Vero E6 cells were grown to confluency in a 24-well plate and infected in triplicate with VSVwt, rVSV-EΔM-tM2e, or rVSV-EΔM-HM2e at a multiplicity of infection (MOI) of 0.01. After 2 h of incubation, cells were washed and cultured with DMEM 2% FBS. The supernatants were collected at 12, 24, 48, and 60 h post-infection and stored at −80°C. The titers of rVSV in the supernatants were determined by the TCID_50_ method on Vero E6 in a 96-well plate.

### The *Gaussia* luciferase assay

To test the entry ability of PVPs into MDMs and MDDCs, an equal amount (adjusted by P24) of EΔM, EΔM-HM2e, or EΔM-tM2e-GLuc+ PVPs was used to infect MDMs and MDDCs. At various time points after infection, the supernatants from the infected cells were collected and *Gaussia* luciferase (GLuc) activity was measured. Briefly, a 10 μl of the sample was mixed with a 50 μl portion of GAR-1 reagent (Targeting Systems) and then measured in the luminometer (Promega, United States; Ao et al., 2005).

### Immunofluorescence assay

Vero E6 cells were grown on a glass coverslip (12 mm^2^) in a 24-well plate and infected with rVSV-EΔM-HM2e or rVSV-EΔM-tM2e for 48 h. After infection, cells on the coverslip were fixed with 4% paraformaldehyde in PBS for 5 min and permeabilized with 0.2% Triton X-100 in PBS. The cells were then incubated in primary antibodies specific for M2e or HA followed by corresponding secondary FITC-conjugated secondary antibodies. The cells were viewed under a computerized Axiovert 200 fluorescence microscopy (Becto Deckson).

### Mice immunization experiments

**FIGURE 2:**
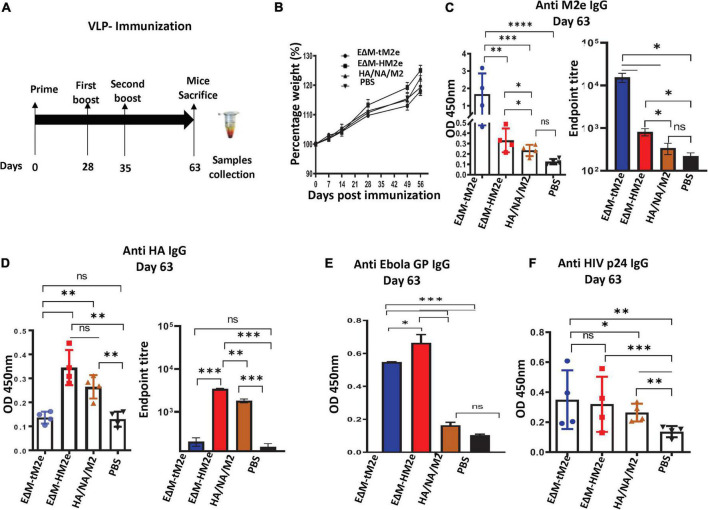
The anti- M2e and anti-HA antibodies were induced by EΔM-tM2e- or EΔM-HM2e -VLPs in Balb/c mice **(A)** Schematic of the EΔM-tM2e, EΔM-HA M2e, or HA/NA/M2-VLP immunization protocol used in this study (*n* = 4/5 mice per group). **(B)** Mice body weights were monitored weekly, in which 100% body weight was set at day 0. **(C)** The levels of anti-M2e, Left panel: OD 450 nm; Right panel: Endpoint titer. **(D)** Anti-HA M2e, Left panel: OD 450 nm; Right panel: Endpoint titer. **(E)** Anti-Ebola GP. **(F)** Anti-HIV P24. IgG-specific antibodies were measured by the ELISA method. Statistical significance was determined using an unpaired *t*-test, and significant *p*-values were represented with asterisks * ≤0.05, ^**^ ≤0.01, ^***^ ≤0.001, and ^****^ ≤0.0001. No significance (ns) was not shown.

Female BALB/c mice aged 4–6 weeks used in this study were obtained from the Central Animal Care Facility, the University of Manitoba (with the animal study protocol approval No. 20-017). For the immunization of VLPs, the mice were divided into four mice per group. On days 0, 28, and 56, mice from each group were immunized subcutaneously by injecting 100 ng (adjusted by HIV p24) of EΔM-tM2e, EΔM-HM2e, and HA/NA/M2 -VLPs in 100 μl of endotoxin-free PBS or PBS only. On day 63, blood samples were collected, and the mice were sacrificed (Figure 2A). For rVSV immunization, mice (five for each group) were immunized intramuscularly (IM) with 1 × 10^7^ TCID_50_ rVSV-EΔM-tM2e or rVSV-EΔM-HM2e and boosted on day 21 with 5 × 10^6^ TCID_50_ of the same rVSV and sacrificed on day 35. Blood samples, nasal wash, and splenocytes were collected on day 35 (Figure 4A).

**FIGURE 3:**
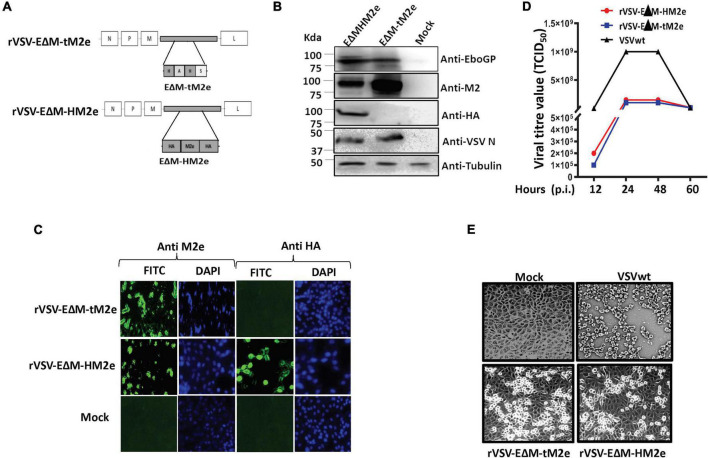
Generation and characterization of rVSV expressing the EΔM-tM2e or EΔM-HM2e. **(A)** Schematic structures of recombinant vesicular stomatitis virus (rVSV-ΔG) vector expressing the EΔM -tM2e or EΔM-HM2e. **(B,C)** The expressions of the EΔM-tM2e, EΔM-HAM2e, EboGP, and VSV-N proteins in the rVSV-infected Vero E6 cells by WB or immunofluorescence assay. **(D)** Vero E6 cell was infected with rVSV-EΔM-tM2e, rVSV-EΔM-HM2e, or VSVwt with MOI 0.01. Supernatants were collected at various time points and were titrated using Reed and Muench method (Gauger and Vincent, 2014). **(E)** At 48 h post-infection, the cells were observed under the microscope.

**FIGURE 4:**
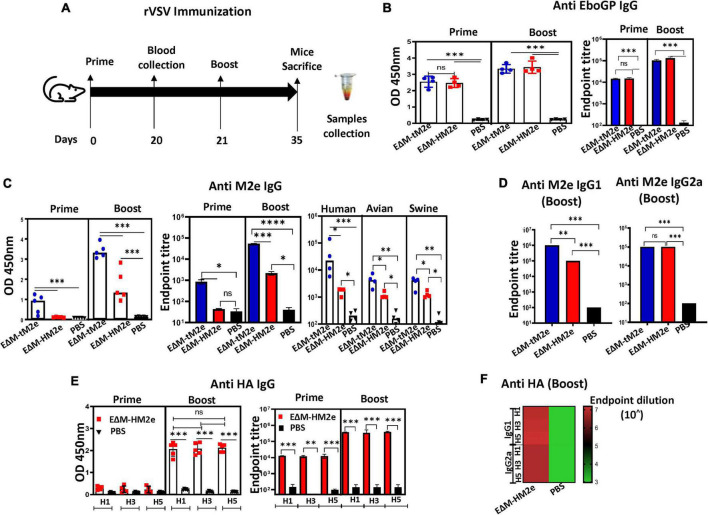
The specific anti-M2e and anti-HA IgG induced by rVSV-EΔM-tM2e or rVSV-EΔM-HM2e in mice sera. **(A)** The Balb/c mice were injected IM with 1.0 × 10^7^ TCID_50_ of rVSV-EΔM -tM2e or rVSV-EΔM-HM2e on day 0 and 5 × 10^6^ TCID_50_ on day 21. Serum was collected on days 20 and 35 (*n* = 4/5 mice per group). **(B)** ELISA assay was used to detect the levels of anti-Ebola GP IgG (Left panel: OD 450 nm; Right panel: Endpoint titer). **(C)** Anti-M2e IgG (Left panel: OD 450 nm; Middle panel: Endpoint titer; Right panel: Test against human, avian, and swine M2e peptides). **(D)** Anti-M2e IgG1 and IgG2a. **(E)** Anti-HA (HA1, HA3, and HA5) IgG (Left panel: OD 450 nm; Right panel: Endpoint titer) and anti-HA IgG1 and IgG2a **(F)**. Statistical significance between the two groups was determined using an unpaired *t*-test, and significant *p*-values are represented with asterisks, * ≤0.05, ^**^ ≤0.01, ^***^ ≤0.001, ^****^ ≤0.0001. No significance (ns) was shown.

For influenza viral challenge in mice, the mouse-adapted strain of A/Puerto Rico/8/34 (H1N1) or H3N2 virus strain was used. Each group of mice (five for each group) was IM immunized with 1 × 10^7^ TCID_50_ of rVSV-EΔM-tM2e, rVSV-EΔM-HM2e, or endotoxin-free PBS and boosted with 5 × 10^6^ TCID_50_ of the same rVSV or endotoxin-free PBS on day 21. Two weeks after the final immunization, the challenge was done by infecting the mice intranasally with 700 or 2.1 × 10^3^ PFU of H1N1 or 1.4 × 10^4^ PFU of H3N2, while mice injected with PBS were challenged as a negative control. Weight loss or gain of the mice was monitored daily for 2 weeks after the challenge.

### Enzyme-linked immunosorbent assay for measurement of antibodies in mouse serum and nasal wash

To determine influenza HA- and M2-specific antibodies in mice sera, ELISA plates (NUNC Maxisorp, Thermo Scientific) were coated with 100 μl of M2e peptide (SLLTEVETPIRNEWGCRCNDSSD) or rHA (H1, H3, or H5) proteins (1 μg or 0.5 μg/ml, respectively) in a coupling buffer (0.05M carbonate-bicarbonate buffer of pH 9.6) overnight at 4°C. To measure the HIV Gag-specific or EboGP-specific antibody in sera, the plate was coated with HIV-1 IIIB p24 recombinant protein (0.5 μg/ml) or recombinant glycoprotein (0.5 μg/ml) as described previously (Ao et al., 2019). The serum samples were diluted with primary antibody diluent (1:100–1:10^9^), and then 100 μl of the diluted mouse serum samples was added into each well of the plates and incubated for 2 h at 37°C. After washing, 100 μl of peroxidase-conjugated goat anti-mouse immunoglobulin G (IgG), IgG1, IgG2a, or IgA secondary antibodies were added and incubated for 1 h at 37°C. Finally, 3’,3’,5’,5’ tetramethylbenzidine (TMB; Mandel Scientific) was added, and the absorbance at 450 nm (OD450) was measured.

### Cytokine detection

Splenocytes from immunized mice were collected aseptically and cultured in 48-well plates at a density of 2 × 10^6^/125 μl with DMEM containing either M2 or HA peptide (1 or 2 μg/peptide/ml), respectively. After 3 days of culturing, supernatants were collected and stored at −80°C for a cytokine detection assay. Cytokine [interferon (IFN-γ), IL-2, IL-4, IL-5, and macrophage inflammatory protein-1 (MIP-1)α] levels were measured using the V-plex mouse MSD V-plex kit (Meso Scale Discovery, United States) following the manufacturer’s instructions.

### Statistics

Statistical analysis of cytokine levels, including the results of GLuc assay, influenza M2e, HA, ELISA, and various cytokine/chemokines, were performed using the unpaired *t*-test (considered significant at *P* ≥ 0.05) by GraphPad Prism 9.3.1 software.

## Results

### Construction and characterization of EΔM-tM2e and EΔM-HM2e chimeric fusion proteins

The HA stalk and M2e remain conserved among the strains of influenza virus and are targets for universal vaccine candidates (Neirynck et al., 1999; Krammer and Palese, 2013; Cummings et al., 2014; Uranowska et al., 2014; Mullarkey et al., 2016; Tsybalova et al., 2018; Krammer, 2019). To elicit a potent and broad immune response, we fused HAs and/or M2e with EboGPΔM (EΔM) that can efficiently target DCs and macrophages. Since there are some amino acid replacements in the avian- or swine-origin influenza viruses (Liu et al., 2005; Kim et al., 2013), we have synthesized four M2e sequences, including conserved human (two copies), swine (one copy), and bird (one copy) M2e by GGG linker and named “tM2e” (Figure 1A). Additionally, we combined a copy of M2e from the human influenza strain with the certain conserved regions of HA stalk from H1, H3, H5, and H7 using a GSA linker (HM2e; Figure 1B). Each HA epitope in the HA stalk sequence was chosen based on their higher conservations among the H5, H1, H3, and H7 sequences. The arrangement of inserting the M2e sequence in the middle of the different HA stalk epitopes (linked with a flexible spacer) would allow the M2e epitopes to be well exposed among multiple epitopes. The tM2e or HM2e gene sequences were inserted into the EΔM vector and named as EΔM-tM2e or EΔM-HM2e, respectively, (Figure 1C).

We first produced PVPs that incorporated the EΔM-tM2e or EΔM-HM2e fusion protein and investigated their abilities to enter DCs/macrophages. Briefly, EΔM-tM2e- or EΔM-HM2e-expressing plasmids were co-transfected with an HIV Gag-Pol packaging plasmid Δ8.2 and a multigene (reverse transcriptase/integrase/envelope)-deleted HIV vector (ΔRI/ΔE/Gluc) in HEK-293T cells as previously described (Ao et al., 2008, 2016). In ΔRI/ΔE/Gluc, the *nef* gene was replaced with a Gluc gene that will be expressed and secreted into the cell culture media after infection. Meanwhile, EΔM- and influenza H5N1 HA/NA/M2-pseudotyped lentiviral viral particles (PVPs; Ao et al., 2008) were included as controls. After 48 h of transfection, the cells and supernatants containing PVPs were collected, and the presence of different proteins in cells and PVPs were confirmed using WB with the corresponding antibodies (Figures 1D,E). As expected, the anti-EboGP antibody detected the expression of EΔM, EΔM-HM2e, or EΔM-tM2e fusion proteins (Figure 1D, upper panel, lanes 1, 3, and 4), while M2e in EΔM-HM2e, EΔM-tM2e, or HA/NA/M2 was readily observed in the transfected cells using an anti-M2e antibody (Figure 1D). Notably, the expression levels of M2e in EΔM-tM2e-transfected cells were significantly higher than those in EΔM-HM2e-transfected cells due to the presence of four copies of M2e in EΔM-tM2e. Moreover, HA stalk and HA protein in EΔM-HM2e and influenza HA/NA/M2 samples were well detected by anti-HA antibody (Figure 1D, fourth panel, Lanes 1 and 2). Consistently, we also revealed the presence of the fusion proteins EΔM, EΔM-HM2e, or EΔM-tM2e in PVPs (Figure 1E, Lanes 1 to 3). Overall, these results indicated that EΔM-HM2e and EΔM-tM2e fusion proteins were efficiently expressed and incorporated into PVPs.

### The ability of EΔM-HM2e-pseudotyped viral particle or EΔM-tM2e-pseudotyped viral particle to target macrophages and dendritic cells

The EboGP has been shown to play a critical role during the infection of DCs and macrophages by facilitating viral attachment, fusion, and entry (Alvarez et al., 2002; Ye et al., 2006; Lai et al., 2017; Ao et al., 2019). Therefore, we investigated whether the fusion of influenza-conserved HM2e or tM2e with EΔM would impede or affect the ability of EΔM to enter DCs and macrophages. Briefly, we infected human MDMs or MDDCs with equal amounts (adjusted with HIV p24 levels) of EΔM-tM2e- or EΔM-HM2e-pseudotyped Gluc^+^ PVPs. In parallel, the HA/NA/M2 or EΔM-pseudotyped Gluc^+^ PVPs were used as controls. On different days after infection, the ability of the PVPs to enter the cells was monitored by detecting the Gluc activity in the supernatant of infected cells. As expected, EΔM-tM2e-PVPs and EΔM-HM2-PVPs could effectively enter the DCs and macrophages (Figures 1F,G), suggesting that the fusion of influenza HA stalk and/or M2e with EΔM did not affect the cell entry efficiency. Indeed, the highest cell entry was observed in EΔM-HM2e-PVPs, which was probably because the HA from H5N1 also has a good affinity with DCs due to its high glycan constituents (Liu et al., 2016).

### EΔM-HM2e-virus-like particles or EΔM-tM2e-virus-like particles immunization induced a high titer of specific anti-influenza hemagglutinin and M2 antibodies in mice

Since EΔM-HM2e or EΔM-tM2- PVPs significantly target DCs and macrophages *in vitro*, we next investigated whether EΔM-HM2e- or EΔM-tM2e-VLPs strongly stimulated influenza HA and M2e immune responses *in vivo*. For this experiment, we subcutaneously immunized Balb/c mice with equal amounts (100 ng of HIV p24) of EΔM- HM2e-, EΔM- tM2e-, or HA/NA/M2-VLPs (Figure 2A). The body weights of the mice were monitored, and no statistically significant differences were observed (Figure 2B). On day 63 post-immunization, sera from mice were collected and the anti-M2e- and anti-HA-specific humoral responses were determined using ELISAs in plates coated with M2e peptides or recombinant HA from H5N1. Interestingly, our results revealed that immunization of EΔM-tM2e- and EΔM-HM2e-VLPs elicited robust production of influenza M2e-specific antibodies, while EΔM-HM2e-VLPs also mediated significantly higher anti-HA antibody than HA/NA/M2-VLPs on day 63 (Figures 2C,D). Interestingly, EΔM-tM2e induced 4-fold higher anti-M2e than EΔM-HM2e-VLPs in the mouse. Collectively, these results indicate that EΔM-tM2e- and EΔM-HM2e-VLP immunization resulted in significantly stronger specific humoral antibodies against influenza M2e and/or HA.

Given that both EΔM-tM2e- and EΔM-HM2e-VLPs contain EboGP and the HIV-1 Gag protein, we therefore examined the anti-EboGP- and anti-HIV P24-specific humoral immune responses in immunized mice. As expected, we observed a significantly strong anti-EboGP and anti-HIV p24 antibody titers in the sera of mice immunized with EΔM-tM2e- and EΔM-HM2e-VLPs (Figures 2E,F). These observations indicate that the presence of EΔM in the VLPs, indeed, enhanced the immunogenicity of EΔM-tM2e- and EΔM-HM2e-VLPs. Since some limitations are associated with PVP production, including time consumption, cumbersome methodology, and cost-ineffectiveness (Thompson et al., 2013), we therefore further investigated whether the expression of EΔM-tM2e or EΔM-HM2e in the rVSV vector would induce strong immune responses against the influenza virus.

### Construction and characterization of recombinant vesicular stomatitis virus expressing the EΔM-tM2e or EΔM-HM2e chimeric protein

The rVSV vector represents a safe and potent vaccine development platform for stimulating both innate and adaptive immune responses (Geisbert and Feldmann, 2011; Fathi et al., 2019). To construct rVSVG-EΔM-tM2e or EΔM-HM2e, the genes encoding EΔM-tM2e or EΔM-HM2e were inserted into an rVSVΔG vector at the position of VSV-G gene, termed as rVSV-EΔM-tM2e or rVSVΔG/EΔM-HM2e (Figure 3A). Above rVSV were rescued in Vero E6 cells *via* reverse genetics technology (Whitt et al., 2016), and the expression of EΔM-tM2e, EΔM-HM2e, or VSV nucleoprotein (N) in the infected Vero E6 cells was examined by WB (Figure 3B) or immunofluorescence assay (Figure 3C). Our results showed the expression of the M2e protein in rVSV-EΔM-tM2e- and rVSV-EΔM-HM2e-infected cells and HM2e in rVSV-EΔM-HM2e-infected cells.

The replication of rVSV-EΔM-tM2e or rVSV-EΔM-HM2e was evaluated in Vero E6 cells. Briefly, Vero E6 cells were infected with rVSV-EΔM-tM2e, rVSV-EΔM-HM2e, or VSVwt at a MOI of 0.01. At 12, 24, 48, and 72 h after infection, supernatants were collected, and virus titers were measured. The results revealed that VSVwt grew rapidly and reached a titer of 1 × 10^9^ TCID_50_ at 24 h post-infection (Figure 3D), while both rVSV-EΔM-tM2e and rVSV-EΔM-HM2e grew slowly and reached a peak titer of 10^8^ TCID_50_ at 48 h post-infection. Moreover, rVSV-EΔM-tM2e and rVSV-EΔM-HM2e induced mild cytopathic effects on Vero E6 cells compared to the VSVwt (Figure 3E). Together, the above observations demonstrated the attenuated replication ability of both rVSV-EΔM-tM2e and rVSV-EΔM-HM2e.

### The recombinant vesicular stomatitis virus expressing the EΔM-tM2e or EΔM-HM2e fusion protein induced robust anti-M2e and anti-hemagglutinin immunoglobulin G antibody responses in immunized mouse serum

The immune responses induced by rVSV-EΔM-tM2e and rVSV-EΔM-HM2e were investigated *in vivo* by IM immunizing Balb/c mice with 1 × 10^7^ TCID_50_ of rVSV-EΔM-tM2e or rVSV-EΔM-HM2e following a boost (5 × 10^6^ TCID_50_) on day 21 (Figure 4A). The sera from immunized mice were collected on days 20 and 35 post-immunizations, and the anti-EboGP antibody responses present in the sera were detected using ELISA. Both the rVSV-EΔM-tM2e- and rVSV-EΔM-HM2e-immunized groups exhibited a high level of anti-EboGP antibodies (Figure 4B), indicating that immunizations with rVSV-EΔM-tM2e and rVSV-EΔM-HM2e exhibited similar potency for anti-EboGP immune responses.

Importantly, a high titer of anti-M2e IgG antibodies was detected in the sera of the mice immunized with rVSV-EΔM-tM2e, particularly after the boost immunization (Figure 4C, left panel). As expected, the anti-M2e IgG level in the rVSV-EΔM-HM2e-immunized mice was 2- to 3-fold lower than that in the rVSV-EΔM-tM2e-immunized mice (Figure 4C, middle panel). We also observed significantly high anti-M2e titer against avian M2e or swine M2e, indicating the possible cross-protection against influenza from different host range (human, avian, and swine; Figure 4C; Right panel). The anti-M2e IgG subsets present in the total IgG titer were also quantitated by analyzing the T-helper type 1 (Th1)-dependent antibody (IgG2a) and T-helper type 2 (Th2)-dependent antibody (IgG1) immune responses. Interestingly, while both rVSV-EΔM-tM2e and rVSV-EΔM-HM2e induced similar levels of anti-M2e IgG2a antibodies (Figure 4D, left panel), the anti-M2e IgG1 level in the sera of rVSV-EΔM-tM2e-immunized mice was significantly higher than that in the sera of rVSV-EΔM-HM2e-immunized mice (Figure 4D, right panel).

The anti-HA (including H1, H3, and H5) antibody titers in rVSV-EΔM-HM2e-immunized mice were also evaluated. The results revealed that rVSV-EΔM-HM2e immunization induced robust anti-HA-specific IgG against recombinant HA derived from H1N1, H3N2, and H5N1 (Figure 4E). Meanwhile, analyses of IgG subsets revealed that rVSV-EΔM-HM2e induced similar levels of anti-HA IgG1 and IgG2a against H1, H3, and H5 (Figure 4F; Uranowska et al., 2014; Whittle et al., 2014). Taken together, rVSV-EΔM-tM2e or rVSV-EΔM-HM2e immunization induced robust production of specific anti-M2e and/or anti-HA IgG antibodies in mice.

### Serological and mucosal IgA and cellular immune responses elicited by recombinant vesicular stomatitis virus expressing EΔM-tM2e or EΔM-HM2e.

**FIGURE 5:**
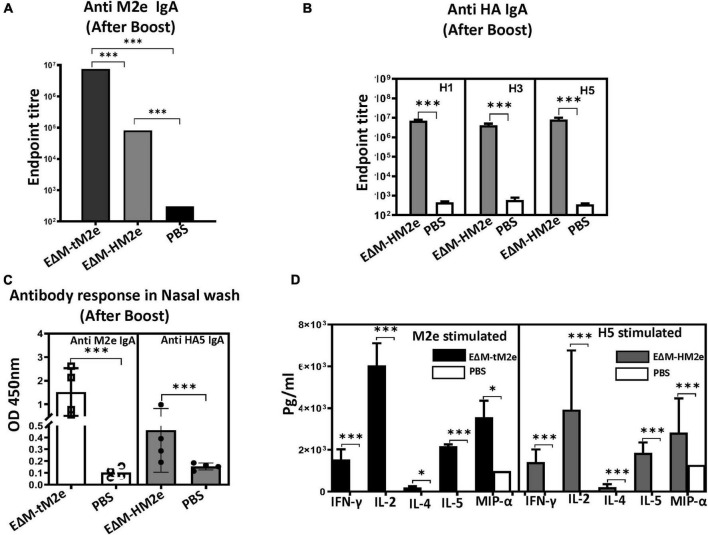
The rVSV EΔM-tM2e or EΔM-HM2e induced specific serological and mucosal Anti-M2e or anti-HA IgA antibodies and mediated cell immune responses. **(A)** The levels of Anti-M2e IgA antibody in mice sera. **(B)** The levels of anti-HA (H1, H3, or H5) IgA antibody in mice sera. **(C)** The levels of Anti-M2e and Anti-HA IgA in the nasal wash. **(D)** Splenocytes were isolated from the rVSV-EΔM-tM2e- or rVSV-EΔM-HM2e-immunized mice and were stimulated with Influenza M2e peptide or HA5 recombinant protein. The release of cytokines and chemokines in the supernatants of cell cultures were quantified with an MSD V-plex kit mouse cytokine kit and counted in the MAGPIX instrument. Statistical significance between the two groups was determined using an unpaired *t*-test, and significant *p*-values are represented with asterisks, * ≤0.05, ^***^ ≤0.001. No significance (ns) was not shown.

Previous studies have shown that robust serological and mucosal IgA responses are associated with a better prognosis of flu disease and decreased influenza transmission (Clements et al., 1986; Belshe et al., 2000; Ambrose et al., 2012; Abreu et al., 2020). Therefore, vaccine-induced IgA antibodies are important immune correlates during influenza vaccination. The levels of anti-M2e or anti-HA IgA in the serum and nasal wash of rVSV-EΔM-tM2e- or rVSV-EΔM-HM2e-immunized mice were determined to evaluate the serological and mucosal IgA response. Importantly, high titers of anti-M2e and anti-HA IgA antibodies were detected in the sera of rVSV-EΔM-tM2e- and rVSV-EΔM-HM2e-immunized mice, respectively, (Figures 5A,B). As expected, the sera from mice immunized with rVSV-EΔM-tM2e- had significantly strong anti-M2e immune responses than rVSV-EΔM-HM2e-immunized mice. Similarly, rVSV-EΔM-tM2e or rVSV-EΔM-HM2e also induced the production of mucosal anti-M2e and anti-HA5 IgA antibodies (Figure 5C). The above-mentioned results indicated that rVSV-EΔM-tM2e or rVSV-EΔM-HM2e could induce anti-IgA antibodies in sera and the respiratory tracts of the immunized mice.

Finally, the cell-mediated immune responses in immunized mice were evaluated. For this experiment, splenocytes isolated from rVSV-EΔM-tM2e- or rVSV-EΔM-HM2e-immunized mice were cultured and stimulated with M2e peptides or HA peptides, and the released cytokines and chemokines were quantified using a Meso Scale Discovery (MSD) V-plex mouse cytokine kit, as described in the section “Materials and methods.” Our results (Figure 5D) revealed that both rVSV-EΔM-tM2e- and rVSV-EΔM-HM2e-immunized mouse splenocytes expressed significantly high levels of IFN-γ and IL-2, which are involved predominantly in the cellular immune response. Meanwhile, a low level of IL-4 levels and abundant production of IL-5, which are linked to tissue protection, were detected. Immunization using the two vaccine candidates also resulted in the production of MIP-1α.

### Immunization with either recombinant vesicular stomatitis virus-EΔM-tM2e or recombinant vesicular stomatitis virus-EΔM-HM2e protects mice from lethal H1N1 and H3N2 influenza virus challenge

**FIGURE 6:**
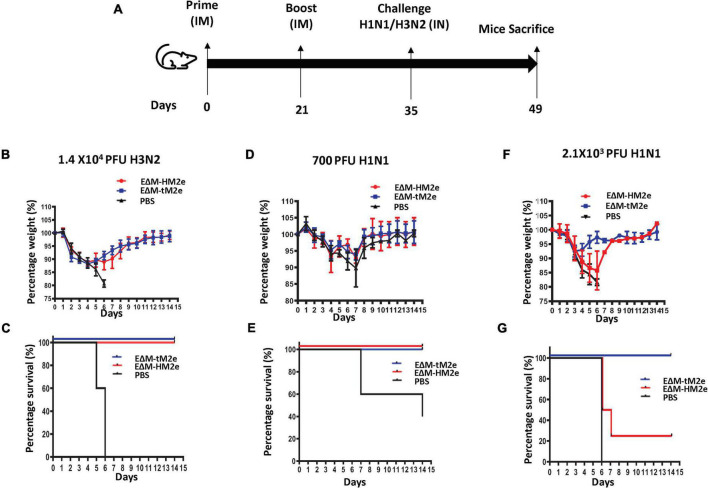
Mice immunized with rVSV-EΔM-tM2e or rVSV-EΔM-HM2e were protected against challenges with H1N1 or H3N2 influenza virus strains. **(A)** The Balb/c mice were injected intramuscularly (IM) with rVSV-EΔM-tM2e, rVSV-EΔM-HM2e, or PBS on days 0 and 14 (*n* = 4/5 mice per group). On day 28 post-immunization, the mice have challenged 1.4 × 10^4^ PFU of H3N2 **(B,C)**; 700 PFU of H1N1 **(E,D)**, or 2.1 × 10^3^ PFU of H1N1 **(F,G)**. **(B,D,F)** Weight loss or gain of the mice. **(C,E,G)** The survival rates of mice after infecting with H1N1 or H3N2.

To determine whether rVSV-EΔM-tM2e- or rVSV-EΔM-HM2e-induced immune responses protected against lethal influenza virus infection, the rVSV-EΔM-tM2e- or rVSV-EΔM-HM2e-immunized mice were challenged with mouse-adapted strain A/Puerto Rico/8/34 (H1N1) or 1968 H3N2 (mouse-adapted A/Hong Kong/1/68) virus (Figure 6A). After the intramuscular administration of rVSV-EΔM-tM2e or rVSV-EΔM-HM2e (primed with 1 × 10^7^ TCID_50_ and boosted with 5 × 10^6^ TCID_50_) or PBS into the mice, we first investigated the protection provided by vaccine candidates against H3N2 influenza virus subtype. The mouse-adapted H3N2 (1.4 × 10^4^ PFU) virus was used to infect the mice. The results revealed that mice vaccinated with rVSV-EΔM-tM2e or rVSV-EΔM-HM2e showed slight weight loss until day 5 and were fully protected (100% survival rate), while the infected control (PBS) group did not survive beyond day 6 (Figures 6B,C).

We then investigated the protection efficacy of rVSV-EΔM-tM2e or rVSV-EΔM-HM2e on the different dosages of the influenza H1N1 challenge. When immunized mice were challenged with a low dose of H1N1 (7 × 10^2^ PFU), the PBS showed a substantial weight loss until day 7 and had a 40% survival rate (Figure 6D). However, both the rVSV-EΔM-HM2e- and rVSV-EΔM-HM2e-immunized groups showed 100% protection with a slight loss of body weight but recovered quickly (Figures 6D,E). While in vaccinated mice challenged with a high dose of H1N1 (2.1 × 10^3^ PFU), morbidity was experienced after the challenge among the rVSV-EΔM-HM2e-vaccinated mice until day 6, as evidenced by a 15–20% loss of body mass with 25% survival rate (Figure 6F), whereas rVSV-EΔM-tM2e-vaccinated mice had 100% protection with moderate weight loss (Figures 6F,G).

All these results provided evidence that immunization with rVSV-EΔM-tM2e efficiently protected mice from the H3N2 influenza challenge. However, in the H1N1 influenza challenge, rVSV-EΔM-tM2e had complete protection against the low and high dosages of H1N1, while rVSV-EΔM-HM2e only had 100% protection against the low dose and partial protection against the high dose of H1N1 influenza challenge.

## Discussion

A flu vaccine that is effective against multiple strains of influenza has been a goal for decades. In this study, we generated VLPs and rVSV-based vaccine candidates expressing the ectodomain of influenza matrix protein (M2e) and/or conserved hemagglutinin stalk regions (HA stalk) with the DC-targeting domain of EboGP (EΔM) and revealed their abilities to efficiently elicit anti-influenza immune responses and protect against different strains of influenza virus.

Recent advances in influenza virus vaccine research have shown that targeting the conserved epitopes in the HA stalk or M2e of the influenza virus is a promising approach for developing influenza A universal vaccine (Uranowska et al., 2014; Mullarkey et al., 2016; Mezhenskaya et al., 2019, 2021). However, methods to enhance the immunogenicity of these polypeptides are still an important issue to be addressed. According to our recent study, insertion of the heterologous large polypeptides into EboGP at the MLD position can efficiently deliver the inserted polypeptides to human macrophages/DCs and induce robust immune responses (Ao et al., 2021). Therefore, in this study, we inserted M2e (four copies) or M2e-HA stalk fusion (aa156) into EΔM, expecting to increase their immunogenicity. Indeed, our study showed that EΔM-HM2e- and EΔM-tM2e-PVPs exhibited a strong ability to target and enter DCs and macrophages, compared to HA/NA/M2-PVPs (Figures 1F,G). The animal study demonstrated that EΔM-tM2e- or EΔM-HM2e-VLPs induced significantly higher anti-M2e and/or anti-HA antibody titers than that induced by HA/NA/M2-VLPs (Figures 2C,D). The anti-p24 antibody titer in EΔM-tM2e-VLPs- or EΔM-HM2e-VLPs-immunized mice was also higher than that in HA/NA/M2-PVP-immunized mice (Figure 2F). As expected, EΔM-tM2e-VLPs resulted in remarkably higher anti-M2e antibody levels than that of EΔM-HM2e-VLP immunization (Figures 2C,D). Overall, the observations provide evidence to support our hypothesis that the presence of EΔM-tM2e or EΔM-HM2e on the surface of VLPs induces efficient antibody responses to M2e and HA.

The rVSV vector has attracted global attention as a vaccine delivery system that can induce strong humoral and cell-mediated immune responses against viral proteins (Majid and Barber, 2006; Furuyama et al., 2020). Interestingly, a recent study showed that rVSV expressing HA stalk of influenza confers rapid protection against different strains of H5N1 serotype (Furuyama et al., 2020). In our study, we replaced VSV glycoprotein with Ebola glycoprotein (EΔM) and resulted in a significantly attenuated replication of rVSV-EΔM-tM2e or rVSV-EΔM-HM2e (Figure 3D), which is important for the elimination of toxicity and pathogenicity of wild-type VSV. Moreover, the robust M2e immune responses elicited by rVSV-EΔM-tM2e or rVSV-EΔM-HM2e presumably are due to the immunogenicity of the VSV vector and Ebola GP DC-targeting domain (EΔM**).** Interestingly, we observed a high titer of IgA and IgG in both serum and mucosal surfaces of the IM-immunized mice. Some previous studies have indicated that although the IgA induced in the serum is from a different plasma cell compartment when compared with the gut or lung mucosal IgA, the serum IgA and the gut or lung IgA share a strong clonal overlap (Iversen et al., 2017; Neu et al., 2019; Abreu et al., 2020; Sterlin et al., 2020), explaining the possible reason for the induction of IgA immune response in the mucosal surfaces. Notably, the early induction of IgA either in the serum or mucosa is important for viral clearance (Sterlin et al., 2020). Moreover, the robust M2e immune responses elicited by rVSV-EΔM-tM2e or rVSV-EΔM-HM2e presumably are due to the high immunogenicity of VSV vector and Ebola GP DC-targeting domain (EΔM).

To increase the immunogenicity of M2e, we designed four copies of M2e (two from humans and one each from avians and swine; Liu et al., 2005) that led to an increase in the amounts of M2e protein on the surface of viral particles and/or the cells. This strategy resulted in significant increases in the production of M2e-specific antibodies and cell-mediated immune responses. Notably, the anti-M2e immune response induced by rVSV-EΔM-tM2e and rVSV-EΔM-HM2e are not only able to bind to human M2e, but also efficiently interact with the avian and swine M2e (Figure 4C, right panel). These findings suggest that vaccination with rVSV-EΔM-tM2e may provide more broad protection against different influenza A infections from various host range (human, avian, and swine). Indeed, rVSV-EΔM-tM2e *via* intramuscular administration provided 100% protection against lethal H1N1 and H3N2 influenza virus challenges (Figure 6). Further investigation is under the way to test whether rVSV-EΔM-tM2e could mediate broad protection against another human influenza A virus and the avian/swine virus infections.

Meanwhile, our study also showed that rVSV-EΔM-HM2e induced high levels of antibody responses to HA from different influenza strains, including H1N1, H3N2, and H5N1 (Figures 4E,F, 5B), indicating its broad anti-HA responses. The rVSV-EΔM-HM2e vaccine immunization also provided 100% protection against challenge with a lethal dose of H3N2 and a low dose of H1N1 but 25% protection in a lethal dose of H1N1 (Figure 6). Since the HA stalk epitopes present in HM2e do not represent the full length of the H5N1 HA stalk (Figure 1B), it is necessary to further select and/or modify the HA part in E?M-HM2e to improve its immunogenicity and induce a stronger neutralization antibody.

Overall, this study demonstrated that rVSV expressing EboGP with influenza HA stalk region and/or M2e fusion protein elicited robust influenza immune responses and protected against different strains of influenza virus. This study also provides convincing evidence for the EboGP-based DC-targeting domain (EΔM) fusion technology to be broadly used to develop vaccine strategies. Given that a universal influenza A vaccine should be effective against all influenza A viruses, regardless of any antigenic mutation or HA/NA subtypes, further studies are required to investigate the protective effects of these candidate vaccines on other strains of influenza virus and non-human primates.

## Data availability statement

The original contributions presented in this study are included in the article/supplementary material, further inquiries can be directed to the corresponding author.

## Ethics statement

The animal study was reviewed and approved by Center for Animal Care Services, University of Manitoba.

## Author contributions

TO and XY conceived of and designed the experiments. XY and HA performed the constructions and rescue of different rVSV viruses and the viral replication characterization. TO, MM, and ZA performed the animal immunization studies. TA and ZA performed the cytokine production analyses and written the initial draft of the manuscript. TO, MM, and DK carried out the animal challenge experiments. All other authors contributed to editing the manuscript into its final form. KC, DK, GK, and XY managed and supervised the work. All authors contributed to the article and approved the submitted version.
